# 

**Published:** 1954-07

**Authors:** 


### Contents

| | | Page |
|---|---|---|
| EDITORIAL | | 69 |
| RESPIRATOR RESEARCH AT HAM GREEN HOSPITAL | | |
| CHANGES IN THE EDITORIAL COMMITTEE | | |
| DAMNED DRUGS (PART I) | *Dr. G. E. F. Sutton* | 71 |
| PRACTICAL POINTS IN THE HOME TREATMENT OF TUBERCULOSIS | *Dr. C. de W. Kitcat* | 79 |
| THE TROPICS ON OUR DOORSTEP | *Sir George R. McRobert* | 83 |
| COLONIAL MEDICAL RESEARCH | *Prof. H. S. L. Heller* | 91 |
| A RADIOLOGIST VISITS AFRICA | *Dr. J. H. Middlemiss* | 95 |
| MONGOLISM, HYPERTROPHIC PYLORIC STENOSIS TREATED WITH "SKOPYL" AND DEATH FROM MILIARY TUBERCULOSIS AND MENINGITIS | | 97 |
| ANNOTATION | | 100 |
| BUNGALOWS FOR DISABLED PERSONS | | |
| NOTES AND NEWS | | 100 |
| EXAMINATION RESULTS | | |
| PRIZES | | |
| APPOINTMENTS | | 101 |
| BOOK REVIEW | | 102 |

